# THE ASSOCIATIONS BETWEEN THE SOCIAL NETWORK, THE CARE NETWORK, AND THE WELL-BEING OF OLDER ADULTS IN THE NETHERLANDS

**DOI:** 10.1093/geroni/igae098.0118

**Published:** 2024-12-31

**Authors:** Marjolein Broese Van Groenou, Norah Keating

**Affiliations:** Chair; Discussant

## Abstract

The increased demand for long-term care (LTC) and the drastic reforms of the LTC systems in western societies create uncertainty among families. It raises the question of whether and how families can provide appropriate LTC for their older relatives and maintain the wellbeing of the care recipient (CR). One solution is for core family members (partners and children) to organize care together with other informal and formal caregivers as much as possible in large and diverse care networks. Well functioning care networks could then possibly contribute to the wellbeing of the CR. The first contribution of this symposium focuses on the informal care potential of the older care recipient and investigates which characteristics of the social network are associated with a care network with many informal helpers. The second paper examines how different types of (informal) care networks contribute to CR’s psychological well-being, and to what extent loneliness, perceived control and care sufficiency play a role in this. The third paper links the theoretical models of both papers and investigates whether and how the associations between the social network, the care network and wellbeing vary in different societal and LTC contexts. All contributions use the Longitudinal Aging Study Amsterdam (LASA), a long-term study into the functioning and care use of older people in the Netherlands. Data are used from ten three-yearly waves between 1992 and 2022. The discussion focuses on which directions the studies provide for improving long-term care for the older care recipients and their informal caregivers.

